# Holistic evaluation of biodegradation pathway prediction: assessing multi-step reactions and intermediate products

**DOI:** 10.1186/s13321-021-00543-x

**Published:** 2021-09-03

**Authors:** Jason Y. C. Tam, Tim Lorsbach, Sebastian Schmidt, Jörg S. Wicker

**Affiliations:** 1 School of Computer Science, University of Auckland, Private Bag 92019, 1142 Auckland, New Zealand; 2 enviPath UG & Co. KG, Postfach 230062, 55051 Mainz, Germany; 3 Bayer AG, Crop Science Division, Environmental Safety, Alfred-Nobel-Straöe 50, 40789 Monheim am Rhein , Germany

**Keywords:** Biodegradation, Metabolic pathways, Machine learning

## Abstract

The prediction of metabolism and biotransformation pathways of xenobiotics is a highly desired tool in environmental sciences, drug discovery, and (eco)toxicology. Several systems predict single transformation steps or complete pathways as series of parallel and subsequent steps. Their performance is commonly evaluated on the level of a single transformation step. Such an approach cannot account for some specific challenges that are caused by specific properties of biotransformation experiments. That is, missing transformation products in the reference data that occur only in low concentrations, e.g. transient intermediates or higher-generation metabolites. Furthermore, some rule-based prediction systems evaluate the performance only based on the defined set of transformation rules. Therefore, the performance of these models cannot be directly compared. In this paper, we introduce a new evaluation framework that extends the evaluation of biotransformation prediction from single transformations to whole pathways, taking into account multiple generations of metabolites. We introduce a procedure to address transient intermediates and propose a weighted scoring system that acknowledges the uncertainty of higher-generation metabolites. We implemented this framework in enviPath and demonstrate its strict performance metrics on predictions of in vitro biotransformation and degradation of xenobiotics in soil. Our approach is model-agnostic and can be transferred to other prediction systems. It is also capable of revealing knowledge gaps in terms of incompletely defined sets of transformation rules.

## Introduction

Data requirements for environmental risk assessments of chemicals are rapidly increasing, for example in regulatory processes at the European (cf. REACH [1]) and global level, but also for the development of new chemical products with more benign profiles. This includes increasing knowledge about transformation products of these chemicals in the environment and increases the need for prediction methods of metabolism and microbial biotransformation, along with the transformation pathways.

**Fig. 1:**
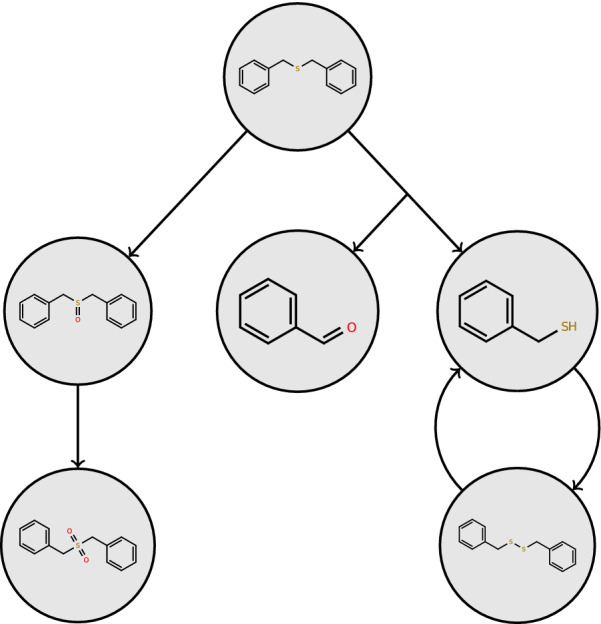
The pathway *Benzyl Sulfide* from the EAWAG-BBD package. Further details of the pathway are available at enviPath [3]

Conceptually, biotransformation pathways represent the chemical changes a given starting compound (referred to as root compound in the remainder of the text) undergoes upon biotransformation. They are constructed from compounds (i.e. molecular structures) connected by reactions. The pathway structure can be represented as nodes and edges in a graph. Figure 1 shows the *Benzyl Sulfide* pathway from EAWAG-BBD (Biocatalysis/Biodegradation Database) [2] as an example.

Existing methods for the prediction of biotransformation products and pathways can be categorized as either knowledge-based, machine learning-based, or hybrid. Each of the two former approaches has its strengths and weaknesses. Knowledge-based approaches use expert knowledge on the basis of sets of transformation rules, in general leading to a combinatorial explosion caused by the overly general nature of the rules. Machine learning-based approaches predict pathways solely based on existing data, the performance being limited by the lack of large data sets.

Hybrid methods, such as machine learning-based relative reasoning models [4–6] predict probabilities of individual transformation reactions by combining knowledge- and machine learning-based approaches. They are constructed using sets of biotransformation pathways and transformation rules as training data, such as the EAWAG-BBD [2] and EAWAG-SOIL [7] packages. These models predict which of the transformation rules that are applicable to a given compound will be correct for that compound.

Biotransformation or metabolism studies from laboratory experiments or environmental observations are the basis for both training and evaluating these models and usually report only transformation products that were formed in high quantities. This is because low concentration metabolites are considered less relevant and are more difficult to analyze and identify. Therefore, metabolites formed in low concentrations are less likely to be reported. This becomes more relevant for higher generation metabolites, because pathways typically diverge into multiple branches and transformations occur on different time scales. Both effects lead to decreasing maximum concentrations with increasing depth in the pathway. Thus, uncertainty about the actual formation of unreported metabolites increases for higher generation metabolites.

The performance of prediction models is typically determined by comparing the predicted transformations for each individual compound against the associated transformation products in the experimental reference pathway. This approach does not take into account the position of the compound or reaction in the pathway. Problems arise when:

- Multi-step reactions are represented as a single step in the experimental data.
- Intermediate metabolites are not observed or not elucidated.
- Transformation products are incorrectly assigned to the wrong educt.
- Concentrations of downstream metabolites become too low to be observed.
- Rule-based evaluation systems fail to address observed transformations not covered by the transformation rules.

In this paper, we address these shortcomings by introducing a new *Multi-Generation* evaluation approach that addresses some of the problems of the state-of-the-art *Single-Generation* evaluation approach. *Multi-Generation* evaluation explicitly includes the compound positions in the graph. Instead of only comparing the reactions for each compound independently, entire predicted pathways are compared to experimentally derived validation pathways. Predictions at higher depth get reduced weights to account for the increased uncertainty due to higher likelihood of minor transformation products not being reported in the experimental reference pathway.

The new evaluation approach further introduces a way to treat intermediate metabolites in the predicted pathway. These metabolites are quickly transformed to downstream products and therefore exist only in very low concentrations. As a consequence, they are often neglected or not analyzed in experimental reference pathways. *Single-Generation* evaluation approaches tend to incorrectly penalize prediction of these intermediates. However, the new *Multi-Generation* approach can take them into consideration when the downstream products are known. Their prediction is not penalized during the scoring process, and the depths of other downstream compounds in the pathway are adjusted accordingly.

With our new evaluation approach we evaluate whole pathway predictions more realistically than before, independent of the underlying set of transformation rules, more in line with the expectations of experimentalists, and more comparable across models. Therefore, we propose to consider the pathway holistically upon evaluation of predictive performance.

Overall, our main contributions are: (1) A new scoring system that quantifies the agreement between two biotransformation pathways. (2) Consideration of compound position (pathway depth) information in the evaluation of pathway predictions via scoring weights. (3) Consideration of intermediate metabolites in the evaluation of pathway predictions. (4) Use of conditional probabilities for depth considerations in predicted biotransformation pathways. This will enable further improvements of the prediction models in future work. Our methodology is a special case of graph analysis that is particularly useful for (bio)degradation or metabolic pathways and chemical reaction networks.

### Background & related work

In this section, we will first give an overview of state-of-the-art prediction systems for biodegradation pathways and their methods. Then we will summarize related work to our proposed evaluation of prediction systems for biodegradation pathways.

#### Biodegradation

Biochemical Network Integrated Computational Explorer (BNICE) [8] is a framework that generates all known reactions for compounds. It uses the set of enzyme reaction rules based on the enzyme commission (EC) classification system. BNICE generates metabolic pathways by first determining functional groups contained in the root compound, and then generates associated products if the reaction rules are applicable. The process is repeated on each of the products in successive generations. The iteration terminates when a threshold is reached, or when no new compounds are created.

METEOR [9] provides the option of knowledge based prediction methods as well as machine learning approaches. The knowledge based option utilizes a combination of *Absolute* and *Relative Reasoning* in their predictions of reactions. The process commences by applying biotransformation rules to the starting compound, and these generate potential metabolites. The absolute reasoning process then assigns a level of belief to each biotransformation [10, 11]. Biotransformations that satisfy the absolute reasoning threshold preset by the user are then ranked in the relative reasoning process. The process uses a relative reasoning threshold to calculate the resulting relative hierarchy. *Static Scores* and *Site of Metabolism Scoring* are other prediction options that make use of machine learning techniques. The first utilizes an occurrence ratio—actual occurrences over all possible occurrences. The latter further considers similarity on additional chemical properties—attributes from generated fingerprints and molecular weights. The processes in each of these options are repeated for all surviving biotransformations, until some preset stopping conditions are satisfied, such as reaching the maximum depth.

PathPred [12] executes predictions by first searching for compounds from the KEGG [13] COMPOUND database that are similar to the chosen starting compound. The results are then used as input to search through the KEGG REACTION database for matching RDM transformation patterns [14]. These patterns are defined as KEGG atom type changes at the reaction center (R), the difference region (D), and the matched region (M). Products of these matching reactant pairs are then used as input, and this process is repeated until stopping conditions are reached. The Jaccard coefficient between the query and matched compounds of each reaction is used as the *reaction score* to indicate its plausibility. The average of all individual reaction scores in the pathway gives the *pathway score*.

EAWAG-PPS (formerly UM-PPS) [2] performs pathway prediction by first determining the functional groups in the starting compound, and applies biotransformation rules to determine the transformed products. Applying these rules iteratively to the educts would lead to combinatorial explosion, and known pathways were used to determine biotransformation priorities [15]. User input is used at the end of each transformation prediction, to determine whether prediction continues downstream of the predicted compound(s). The predicted pathway grows as this cycle is repeated.

Biotransformer [16] combines a rule or knowledge based approach in conjunction with a machine learning approach, to predict metabolic reactions for compounds. It makes use of experimentally confirmed biotransformations derived from the literature, as well as precedence rules that were derived from reported observations. Many of them are from the EAWAG-PPS database. The Biotransformer Metabolism Prediction Tool (BMPT) then uses a set of random forest and ensemble prediction methods to predict reactions, for example related to Cytochrome P450 enzymes (CYP450) and Phase II metabolism. For the latter, a simple rule-based filter is applied to eliminate the most trivial non-candidates for a few chemical classes with known metabolism. Metabolic pathways are predicted progressively starting from the root compound, one reaction at a time.

OASIS TIMES [17] predicts chemical toxicity by integrating metabolism simulators into models assessing toxicity of both the transformation educts and products. This has improved model performance significantly compared to traditional approaches that don’t consider metabolic transformation products. The incorporated metabolic logic accounts for enzyme interactions, channeling effects and depletion of highly reactive intermediates. The metabolism simulator aims to correctly reproduce experimentally observed metabolites, and uses xenobiotic pathway data from MetaPath [18] as a reference and aims to reproduce the observed pathways. However, it simulates metabolism using a complex mathematical model rather than a rule-based approach.

#### Pathway evaluation and comparison

Integrated scoring systems that attempt to quantify the quality of predictions are not always found in the systems mentioned above. PathPred [12] computes the Jaccard index on compounds in each of the predicted reactions, and uses the average of all such values in a pathway as the overall score. OASIS TIMES [17] takes the union of the observed and predicted pathways to tally true/false positives/negatives by comparing the metabolites. Only the first false positive in a sequence of false positives would be penalized, because the rest are conditioned from it. The system can also identify intermediates, and either reward, penalize or ignore them, based on a user-defined parameter. Prediction performances in published work for systems such as METEOR, BNICE and Biotransformer are obtained only from independent tests, without integrated options to evaluate the quality of predictions on new test sets.

A related field is the prediction of graph networks using machine learning techniques. Link prediction is a core component in many of the different approaches, such as analyzing information directly from the graph. This includes common neighbors [19], using metadata of the nodes from the application domain [20], or making use of pre-existing information on the connections between nodes in the graph [21]. There are similar concepts in these approaches and the work in this paper, and we will explore them further for applicability in future work.

Research in Graph Isomorphism addresses the quantification of similarity between graphs. Many techniques focus on properties such as orientation or structural arrangements that share little relevancy with biotransformation pathways. However, common metrics such as Graph Edit Distance [22] can be useful in potential scoring systems or comparing predicted and observed pathways. Nevertheless, in biotransformation studies, the resulting pathways are tentative manual assignments by experts. They do not always reflect the absolute ground truth of the underlying reaction mechanism.

In summary, the work related to predicting biodegradation pathways so far does not take pathway structures into account. Our work in this paper aims to fill this gap by introducing a new approach that evaluates the predictions accordingly.

## Methods and experiments

In this section, we will first summarize the prediction and evaluation of models in enviPath [6], and then introduce our new *Multi-Generation* evaluation that overcomes the limitations of the current approach.

### Single-Generation evaluation

The standard enviPath Relative Reasoning models [4–6, 15] use a chosen set of biotransformation pathways as training data. The set of biotransformation rules consists of rules that were curated by experts. All compounds in the training pathways are independently cross-referenced with the rules for their applicability, producing effectively a quasi Boolean Matrix [23] that describes their inter-relationships. The matrix connects the compounds and rules in a manner similar to: 

with rules $r_n$ and compounds $c_n$ with $n = [1,...5]$ in the training. The values in the matrix elements represent *Not applicable* (− 1), *Applicable but not observed* (0) and *Applicable and observed* (1). A machine learning model is trained on this matrix and then later used to predict probabilities for the combination of a new compound and the set of transformation rules.

In the *Single-Generation* evaluation, the predictions are then compared on the level of single reactions to the ground truth. That is, the known transformations are matched to the predictions for each rule and translated into *true positives*, *true negatives*, *false positives*, or *false negatives*. These counts then are translated to standard performance measures such as accuracy, recall, or precision. Multi-label approaches are used to aggregate the single transformation rule performance to one measure for the whole model. A detailed overview of the training and evaluation process is given in previous work [4].

### Multi-Generation evaluation

In contrast to the procedure outlined above, our *Multi-Generation* approach does not operate on the transformations of each individual compound, but first predicts a whole pathway (see next section) and then operates on the compounds as nodes in the graph. This leads to a couple of additional aspects that require consideration. Please note, however, that the underlying prediction model is identical in both approaches.

As discussed before, compounds in the first generation naturally carry higher confidence in the experimental findings, compared to transformations occurring at higher depth in the pathway. This is due to the amount of test substance being divided into multiple reaction branches and only slow conversion over time. Thus, concentrations of higher-generation products are lower, which makes them more difficult to confirm experimentally and less likely to be reported. We therefore introduce a scoring system within our approach to account for the increasing uncertainty when comparing predicted and observed pathways.

This scoring system assigns rewards and penalties with weights according to the generation of the respective compounds. The resulting score for a pathway represents the agreement between the predicted and observed pathways. The collective scores for each of the pathways in the validation set are used to compute conventional metrics such as recall-precision curves. This new approach evaluates the pathway as a whole across multiple generations of compounds. This is in contrast to approaches in previous work where predicted reactions in each single generation are evaluated independently.

The prediction quality of Relative Reasoning models depends on the compatibility between the transformation rules and the training set, as well as the test set. Rule sets with low compatibility can lead to problematic scenarios, e.g. where no applicable rules can be applied to the target compound structure. In the *Single-Generation* evaluation process, such scenarios would result in all (if any) observed reactions from the educt being ignored. However, if there are further reactions for the product compound in the data, they would still be evaluated. Alternatively, in the *Multi-Generation* evaluation approach, the prediction would terminate at the initial educt and no further scores will be rewarded besides false negatives for the observed products.

**Fig. 2:**
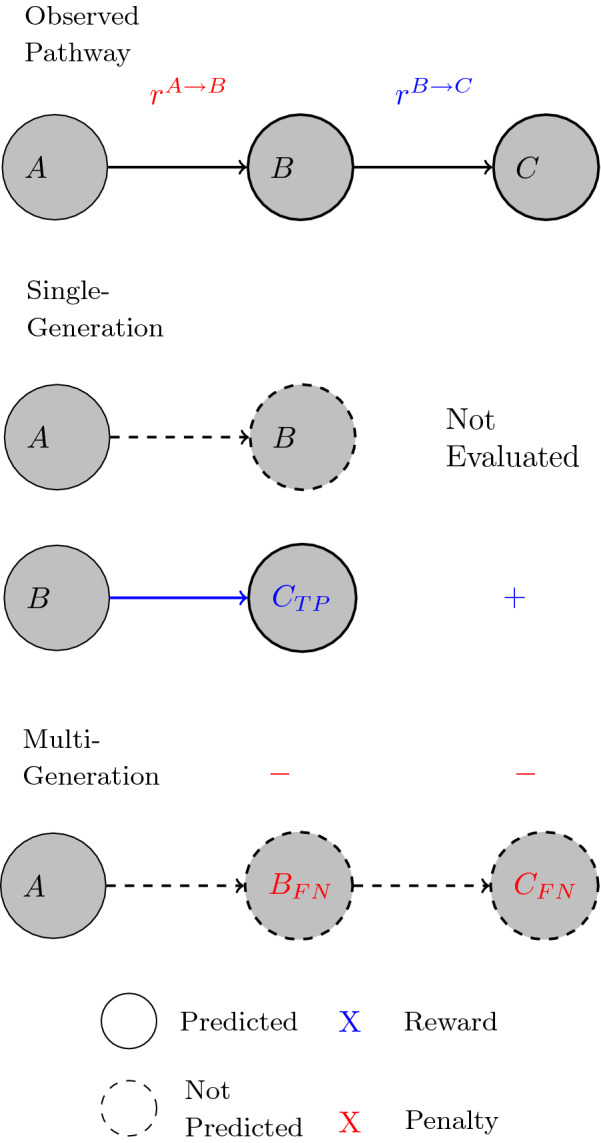
A scenario where a reaction from an observed pathway is not described by any transformation rule used for training. The observed pathway has compound *A* transformed to *B* then to *C*, with the reaction from $B\rightarrow C$ described by a transformation rule ( $r^{B\rightarrow C}$) but none for $A\rightarrow B$ ( $r^{A\rightarrow B}$). The *Single-Generation* evaluation approach would only evaluate $B\rightarrow C$ (with a reward +) and ignore $A\rightarrow B$, since no rule can be applied. The *Multi-Generation* evaluation approach would penalize both compounds *B* and *C* (−) for not being predicted

Figure 2 demonstrates this difference between the two evaluation approaches with a simple example, an experiment that begins with compound *A*. The observed pathway has compound *A* transformed to *B* then to *C*, with the reaction from $B\rightarrow C$ described by a transformation rule ( $r^{B\rightarrow C}$) but none for $A\rightarrow B$ ( $r^{A\rightarrow B}$). The *Single-Generation* evaluation approach would only evaluate $B\rightarrow C$ (with a reward +) and ignore $A\rightarrow B$, since no rule can be applied.

The *Multi-Generation* evaluation approach assigns penalties to both compounds *B* and *C* for not being predicted in the pathway. Although the model assigns a high probability to reaction $B\rightarrow C$, the missing transformation rule for reaction $A\rightarrow B$ prevents any progression along that path. This puts strong emphasis on the knowledge gap in the set of transformation rules and provides a more realistic evaluation metric for the prediction accuracy on the overall pathway level.

#### Multi-Generation pathway construction

We predict pathways in the test or validation set starting from their root compound. Each of the possible reactions is predicted using the supplied transformation rules, which can be represented as a possible branch evolving from the educt. We then calculate conditional probabilities for reactions according to their position in the pathway.

**Fig. 3:**
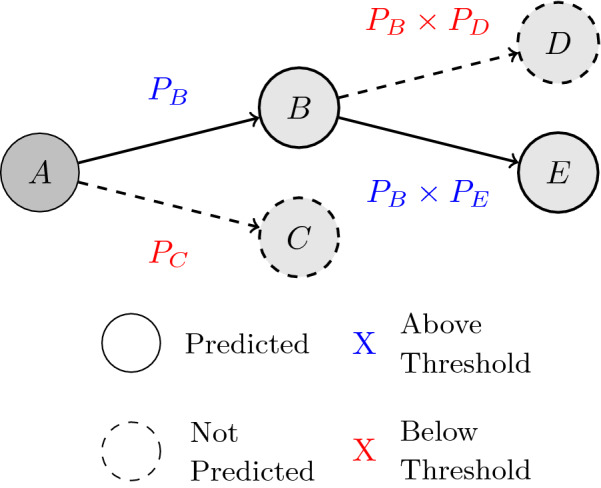
The prediction process for an example pathway. $P_B$ and $P_C$ are probabilities of reactions that would transform compound *A* to compounds *B* and *C*, respectively. $P_D$ and $P_E$ are probabilities of reactions that would transform compound *B* to compounds *D* and *E*, respectively. A hypothetical probability threshold *x* is used to demonstrate how compounds *C* and *D* are pruned from the pathway

This procedure takes into account the relationships between the probabilities of upstream reactions with the current reaction. We adjust the preset threshold value dependent on the depth and use it in the pruning process with the resulting conditional probability. This conditional probability is defined by the product of the probability value assigned to the current reaction, multiplied with values from all of the upstream reactions. An example pathway beginning from compound *A* is shown in Fig. 3.

The example shows root compound *A* with probabilities $P_B$ and $P_C$, for reactions that transform *A* into compounds *B* and *C*, respectively. A probability threshold *x* of value $P_B> x > P_C$ is used in the example, to demonstrate the scenario where compound *C* is predicted to be not observed. The algorithm then continues to determine the possible reactions for compound *B*, transforming to compounds *D* and *E* at the second generation of the pathway, with respective probabilities $P_D$ and $P_E$.

These values are multiplied with $P_B$, generating conditional probabilities, to obtain the conditional probabilities $P_B \times P_D$ and $P_B \times P_E$. They are then tested against the threshold value adjusted for reactions at second generation, at $x^2$. This part of the example demonstrates the scenario where $P_B \times P_E> x^2 > P_B \times P_D$, and compound *D* is predicted to be not observed. This steers the pathway prediction such that branches with high probabilities will be longer, while less likely branches will be cut earlier. Note that while this will change the predictions of a model, this does not introduce a new prediction approach but rather changes the way we use the prediction in the evaluation and application.

#### Performance calculation

We calculate the pathway prediction performance based on standard true/false positive/negative counts, with the notable difference that we apply a weighting system and account for intermediates as described below. The quantities *TP*, *FP* and *FN* are computed as follows:

- *TP* Compounds present in both predicted and observed pathways count as true positives, with weights according to their depth in the observed pathway.
- *FP* Compounds that only exist in the predicted pathway but not the observed count as false positives, with weights according to their depth in the predicted (adjusted) pathway.
- *FN* Compounds that only exist in the observed pathway count as false negatives, with weights according to their depth in the observed pathway.

These definitions are used with the following *Weighting System* and treatment of intermediate metabolites.

#### Weighting system

**Fig. 4:**
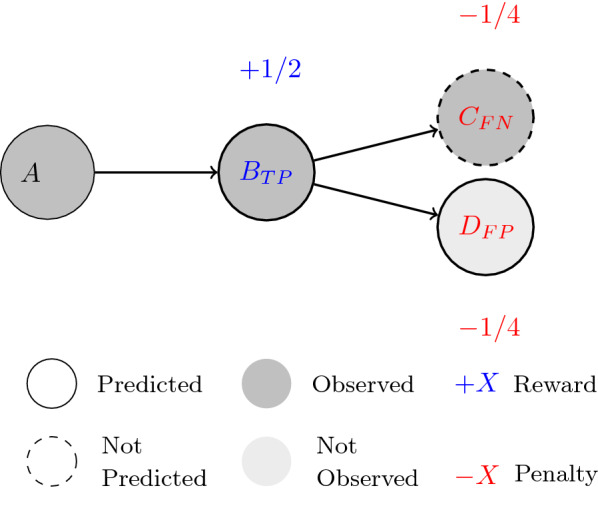
A pathway combined from a prediction and an observed pathway in the comparison process. True/false positives/negatives are determined in the comparison, and weights are assigned according to their depths for rewards and penalty calculations

We propose a mathematical model to compare two pathways with multiple generations. In accordance to the natural decrease in experimental certainty along the pathways, the compounds are assigned decreasing weights as their generation or depth level increases. These weight values start at $\frac{1}{2}$ for compounds at generation or depth level one, and decrease by 50$\%$ for each increasing level. The weights are used as multipliers to conventional classification metrics such as counts of true/false positives/negatives. The multipliers are then used to quantify the agreement between predicted and experimental pathways. We use the *Jaccard Index* as metric for pathway similarity. It is defined as:

$$\begin{aligned} Sim=\frac{\sum (TP\times W_D)}{\sum (TP\times W_D) + \sum (FP\times W_D) + \sum (FN\times W_D)} \end{aligned}$$

where *TP* and *FP* are the *True* and *False* positives, respectively, and *FN* represents the *False* negatives. $W_D$ represents the weight multiplier that is dependent on the depth of the metabolite in the pathway. For an example see Fig. 4. This metric avoids the infinite number of potential true negatives^1^, and gives equal weight to each pathway in the validation set independent of the pathway length. The average score from all pathways in the validation set represents the accuracy of the model.

#### Intermediate metabolites

*Intermediate metabolites* are compounds with enhanced reactivity. They are quickly transformed to downstream metabolites, and therefore exist only in very low concentrations. These intermediates are sometimes included in the experimental data and sometimes not. This depends on the choice of the author of the experimental study report or the data package and the underlying experimental evidence. If they are not included, the transformation of the educt is reported to lead directly to the downstream metabolite. While prediction of such an intermediate would be mechanistically correct, they might not be present in the available data. Such a scenario would incorrectly inflate the count of false positives during the *Single-Generation* evaluation, and would be even more detrimental in the *Multi-Generation* evaluation procedure. The intermediate metabolite would be penalized, along with all metabolites downstream to it, as they would appear at an incorrect depth in the pathway.

In order to correctly accommodate the intermediate metabolites in the evaluation procedure, we have designed a process that adjusts the depth level of the downstream compounds accordingly. The process first determines a list of compounds that are present in both the predicted and observed pathways. Then it checks if any of them are immediately downstream to one another in the observed pathway. The compound pairs which fit this criterion are examined to test if additional compounds are between them in the predicted pathway. These compounds are then added to the list of intermediates. Such intermediate metabolites might still be correctly predicted without the downstream node from the observed pathway. However, the use of a correctly predicted downstream nodes is required to identify them in a reliable manner and treat them properly. In other words, we can correct the evaluation of intermediates if and only if they have downstream products in the reference pathway that were correctly predicted.

The list of intermediate compounds is used to adjust depth levels in the predicted pathway accordingly. The shortest path between each of the compounds in the pathway and the root compound is determined using a *Breadth-first search*. The list of in-between compounds is determined and the depth level of the end compound is then decreased by the number of intermediate compounds that are in this list of in-between compounds. The intermediate compounds are ignored by the *Multi-Generation* evaluation scoring algorithm.

**Fig. 5:**
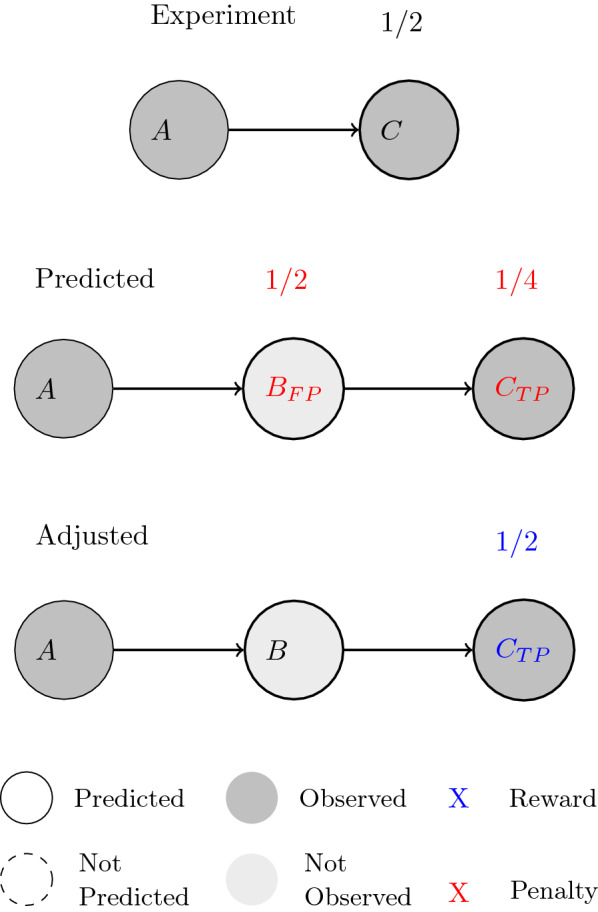
The depth adjustment process according to intermediate metabolites determined in the predicted pathway. Compounds *A* and *C* are present in both observed and predicted pathways, which allows compound *B* to be identified as an intermediate metabolite. It can be ignored and the depth-associated weight for scoring can be adjusted accordingly for compound *C*

Figure 5 shows an evaluation example incorporating concepts from both the *Weighting System* and the treatment of intermediate metabolites.

### Experimental setup

We carried out several experiments to assess the proposed evaluation approach. We used a combination of pathway data selection as well as several experiment designs to evaluate the validity and difference in performance compared to the *Single-Generation* evaluation approach.

#### Biotransformation pathway data

Several sets of biotransformation pathways were used in this work:

EAWAG-BBD The set of biodegradation pathways contained in the EAWAG Biocatalysis/Biodegradation Database package [2] contains primarily xenobiotic chemical compounds and microbial biocatalytic reactions. Information on such microbial enzyme-catalyzed reactions carries great importance in the fields of biotechnology and environmental research.

EAWAG-SOIL The set of biodegradation pathways in the EAWAG-SOIL package [7] contains pesticide degradation pathways compiled from laboratory soil degradation studies. These pesticides are registered in the EU, and their degradation pathways are freely accessible regulatory data.

From the *EAWAG-SOIL* package we selected diverse subsets of pathways as training and test sets that evenly cover the chemical space. This is done to obtain a representative set without over-representation of certain compound clusters. The selection is based on the Tanimoto similarities from Morgan2 fingerprints [24]:

$$\begin{aligned} T(a,b) = \frac{N_{ab}}{N_a + N_b - N_{ab}} \end{aligned}$$

where $N_a$ and $N_b$ are the numbers of 1 bits present in the fingerprints of compounds *a* and *b*, and $N_{ab}$ is the number of 1 bits occurring in both fingerprints. We used the MaxMin algorithm [25] to incrementally pick compounds with the least similarity to the most similar compound from the already selected set. We selected 80$\%$ of the EAWAG-SOIL pathways to become the *TRAIN-SOIL* package for model training purposes. The remaining 20$\%$ make up the *TEST-SOIL* package which is to be used as a test set. We excluded pathways that are not representative for typical organic chemistry, i.e. when their root compounds are inorganic salts, much larger than the rest, or contain heavy metal elements.

#### Experiment designs

We use the set of validated biotransformation rules from the EAWAG-BBD package to build relative reasoning models with compound structures from pathways inside specified training packages. We have set the probability threshold for reactions to a low value of 0.1 for all experiments, in order to efficiently capture differences between the two evaluation approaches. The following are the experiment designs used for examination, and data sets for both evaluation approaches:

Validation Test A procedure to strictly validate the accuracy of the proposed mathematical approach that compares biotransformation pathways. Three sub-procedures are performed:

- *Full pathway* Evaluate each pathway against itself. The result is expected to be 1.
- *Empty pathway* Evaluate each pathway against only its starting compound. As the comparison of the pathway starting compound is ignored in the scoring system, the result is expected to be 0.
- *Half full pathways* A random process is performed to remove all but the starting compound in approximately 50$\%$ of a cloned set of pathways. Each pathway in the original set is evaluated against the associated one in the cloned set. Results of some metrics such as Accuracy and Recall are expected to be close to the ratio of unmodified pathways in the cloned set.

Evaluation with Test Sets A procedure where the entire chosen list of compounds is used to train a relative reasoning model once. Then we carry out the evaluation on the nominated test set TEST-SOIL. This procedure is performed on these pathway set combinations: TRAIN-SOIL, EAWAG-BBD + TRAIN-SOIL.

Evaluation with Holdout This procedure uses a random process to select approximately 66$\%$ of the chosen molecules to train a relative reasoning model. The model is then evaluated on the remaining 34$\%$ of the data. A list of compounds extracted from all selected pathways is used for selection for the *Single-Generation* evaluation approach, and the list of pathways is used for the *Multi-Generation* approach. The process is repeated 100 times, and the results of each individual run are averaged. This approach additionally allows an opportunity to also repeatedly examine the model’s prediction ability on data that is new to the training set. This procedure is performed on these pathway set combinations: EAWAG-BBD, EAWAG-SOIL, TRAIN-SOIL, EAWAG-BBD + EAWAG-SOIL, EAWAG-BBD + TRAIN-SOIL.

## Results and discussion

To assess the effectiveness and validity of our *Multi-Generation* evaluation approach, we summarize the results from the procedures detailed in the *Experiments* section in the following. We calculated Accuracy, Precision, Recall and Area under the Precision-Recall Curve (AUPRC). Due to the nature of the *Multi-Generation* evaluation approach, where pathways potentially have an infinite number of true negatives, the false positive rate cannot be computed. In the *Single-Generation* evaluation approach, the number of true negatives can be calculated from the applicable transformation rules, which are neither predicted (i.e. below the threshold) nor observed experimentally. The Area under the Receiver Operating Characteristic curve (AUROC) is hence only available for the *Single-Generation* approach and is provided as an indicator.

### Illustrative evaluation of an example pathway

**Fig. 6:**
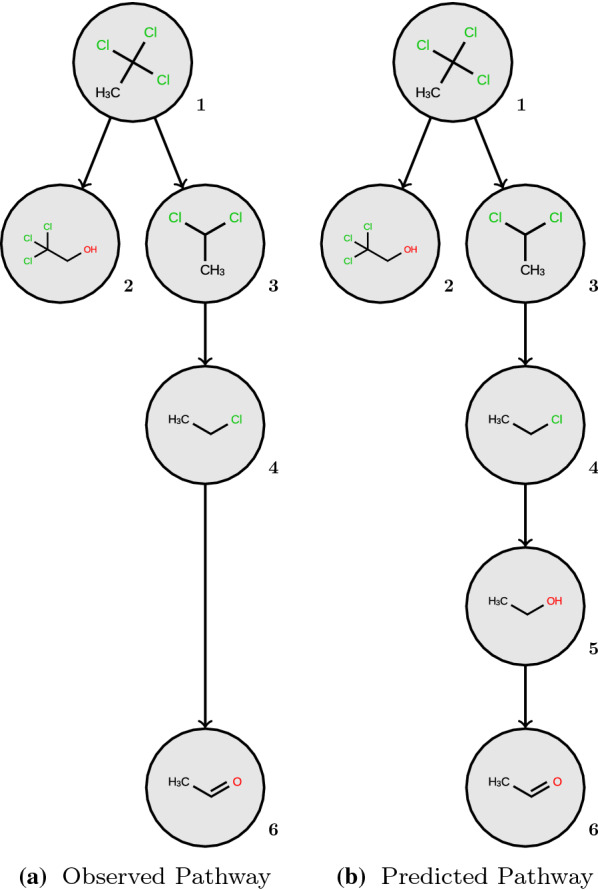
The pathway for *1,1,1-Trichloroethane* as given in the EAWAG-BBD package (**a**), and the corresponding branch of the predicted pathway (**b**)

We demonstrate the main differences between the two evaluation approaches on an illustrative example using the *1,1,1-Trichloroethane* pathway from the EAWAG-BBD package (Fig. 6). For simplicity and better readability, we removed false positive predictions in Fig. 6 and the evaluation below, which would distract from the main points of this demonstration. Please note, however, that five of these false positives are mentioned in the textual description [26] of the pathway as minor products or are reported in the literature (trichloroacetic acid, dichloroacetic acid, ethane, 2-chloroethanol, acetic acid), which highlights the difficulty related to the incompleteness of such minor products in the reference data. The *Multi-Generation* approach mitigates the problem by reduced scoring weights for metabolites at higher depth in the pathway or zero weight for transient intermediate metabolites. Our example demonstrates the impact of not recognizing such intermediate metabolites. It is indicated in the metadata of the observed pathway that the final metabolite acetaldehyde (**6**) is formed indirectly from chloroethane (**4**) via intermediates. Indeed, there is no transformation rule in EAWAG-BBD for this transformation. Therefore, the model predicts acetaldehyde only via ethanol (**5**) or 2-chloroethanol (not shown) as intermediate steps.

In this scenario, the *Single-Generation* evaluation approach will return three true positives: two reactions from the root node leading to products **2** and **3**, and one subsequent reaction from **3** to **4**. The predicted reaction from chloroethane (**4**) to ethanol (**5**) is counted as false positive. The reported transformation from chloroethane (**4**) to acetaldehyde (**6**) is not evaluated at all, because there is no corresponding transformation rule in the underlying set of rules. This example demonstrates that the *Single-Generation* evaluation does not adequately address the likely intermediate and the final product acetaldehyde. The resulting accuracy for this example is 0.75, although the predicted pathway can be considered correct.

The *Multi-Generation* evaluation approach will return two true positives with weight $\frac{1}{2}$ (**2** and **3** at depth = 1), one TP with weight $\frac{1}{4}$ (**4** at depth = 2) and one TP with weight $\frac{1}{8}$ (**6** at depth adjusted from 4 to 3). The intermediate metabolite ethanol (**5**) gets zero weight and does not influence the final score. The resulting *Multi-Generation* accuracy for this example is 1. The comparison of the evaluation metrics from both approaches is summarized in Table 1. Please note that both approaches would yield lower accuracy, if the disregarded false positives would have been included in the example. Thus, ultimately the *Multi-Generation* accuracy would be lower (but better reflect the reality) than the SG accuracy, because it penalizes also the false positives predicted downstream of primary false positives as discussed below.

**Table 1:** Evaluation metrics for the illustrative example *1,1,1-Trichloroethane* from the EAWAG-BBD package

Evaluation approach	Accuracy	Precision	Recall
SG	0.75	0.75	1.0
MG	1.0	1.0	1.0

### Validation tests

Results of the validation tests performed on the EAWAG-BBD compounds are presented in Table 2. As expected, the evaluated full pathways from both packages achieve 1.0 for *Accuracy*, *Precision* and *Recall*, as there are only true positives and no false positives or negatives. The expected values for evaluated empty pathways from both packages are also 0 for all three metrics, as there are only false positives without any true positives. The “Half Full” pathways from both packages achieve 1.0 for Precision, and a value that is proportional to the amount of empty pathways (see Table 3) for Accuracy and Recall. The empty pathways will contribute with false negatives while the full pathways will contribute to the true positive score.

**Table 2:** Results of validation tests performed for Multi-Generation evaluation

Pathways	Accuracy		Precision		Recall	
	BBD	SOIL	BBD	SOIL	BBD	SOIL
Full	1.0	1.0	1.0	1.0	1.0	1.0
Half Full	0.51	0.47	1.0	1.0	0.5	0.47
Empty	0	0	0	0	0	0

The validation process was performed on three different modified versions of the training data itself

**Table 3:** Counts of the full and empty pathways in the validation test process where a random 50$\%$ of pathways are emptied

Pathways	Count	
	BBD	SOIL
Full	113	153
Empty	105	165
Ratio	0.52	0.48

### Evaluation with test sets

**Fig. 7:**
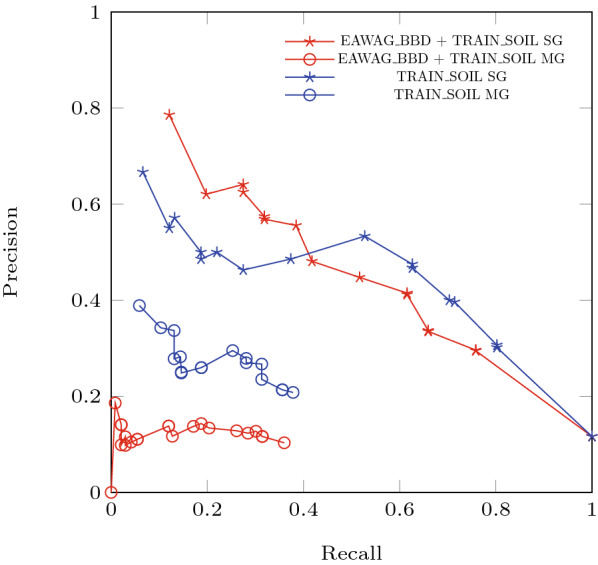
Precision-Recall curves for the *Evaluation with Test Sets* experiments. As the data in the TRAIN-SOIL package is more representative for the evaluated TEST-SOIL package in terms of chemical and biological properties compared to the EAWAG-BBD package, the relative reasoning model trained without the EAWAG-BBD package is more compatible with the evaluation data set. We can see that the *Multi-Generation* evaluation approach better reflects the compatibility between compound structures and the transformation rules used to train the model

Relative reasoning models were trained with the TRAIN-SOIL package and the combination of EAWAG-BBD + TRAIN-SOIL packages. In both cases we evaluated the models on the TEST SOIL package. Tables 4 and 5 show the results, and Fig. 7 gives the associated Precision-Recall curves.

**Table 4:** Statistics of the *Evaluation with Test Sets* experiments for threshold 0.1

Packages	Accuracy		Precision		Recall	
	SG	MG	SG	MG	SG	MG
BBD+TRAIN$\_$SOIL	0.53	0.09	0.34	0.1	0.66	0.36
TRAIN$\_$SOIL	0.6	0.15	0.4	0.21	0.71	0.38

**Table 5:** Statistics of the *Evaluation with Test Sets* experiments for the whole range of thresholds

Packages	AUPRC		AUROC
	SG	MG	SG
BBD+TRAIN$\_$SOIL	0.43	0.04	0.8
TRAIN$\_$SOIL	0.41	0.09	0.82

The numerical values of each metric are noticeably lower for the *Multi-Generation* evaluation approach compared to the *Single-Generation* approach. This has two main reasons: First, the *Single-Generation* evaluation is based only on defined transformation rules, whereas *Multi-Generation* evaluates all nodes in the reference pathway and thus penalizes the incompleteness of the transformation rules. Second, a wrong prediction in the *Multi-Generation* approach is more detrimental, because all the downstream nodes from this branch will be wrong as well. In other words, for a true positive to be tallied, all upstream nodes also have to be predicted correctly. Additionally, a false positive will lead to even more false positives downstream. These two reasons make the *Multi-Generation* approach a much harder evaluation criterion, and thus the lower numerical values do not simply imply a worse result.

Another point worth noting from the *Multi-Generation* evaluation results is that the values for recall do not reach one (see Precision-Recall curve, Fig. 7). The gap between the maximum recall and the value of 1.0 is caused by transformations in the reference pathways which are not covered by transformation rules and their downstream nodes. The products of such reactions can therefore never be predicted correctly, no matter how low the probability threshold is and will always count as false negatives. Moreover, as discussed above, any downstream nodes won’t be predicted either. In contrast, missing transformation rules have no effect on the *Single-Generation* performance, since *Single-Generation* is only evaluated on the existing rules. Thus, the maximum recall value at probability threshold zero can be used as an indicator for the completeness of the rules for the test set.

Furthermore, the results show that the precision values for both approaches do not reach 1, which means the number of false positives does not go down to 0, no matter how high the probability threshold is set. This is expected and implies that there are always products predicted with a high probability that are not correct. Given that the transformation rules are extremely general and can easily be triggered, the role of the machine learning models is to limit this. These results simply show that they do not predict perfectly, which will be hard to achieve with the available data.

The data in the TRAIN-SOIL package is naturally more representative for the evaluated TEST-SOIL package in terms of chemical and biological properties compared to the EAWAG-BBD package. While the EAWAG-BBD+TRAIN-SOIL configuration will provide better predictions for a broader chemical and biological space, the model trained with only the TRAIN-SOIL package will perform better on data that share greater chemical similarity. Therefore, the relative reasoning model trained without the EAWAG-BBD package is more compatible with the evaluation data set. This can be observed in the statistics from the *Single-Generation* evaluation approach. However, the difference is evidently more obvious in the *Multi-Generation* evaluation results, particularly in the Precision-Recall curve. The differences in the areas under the *Multi-Generation* Precision-Recall curves are evidently larger than the *Single-Generation* evaluation counterpart.

### Evaluation with holdout

**Fig. 8:**
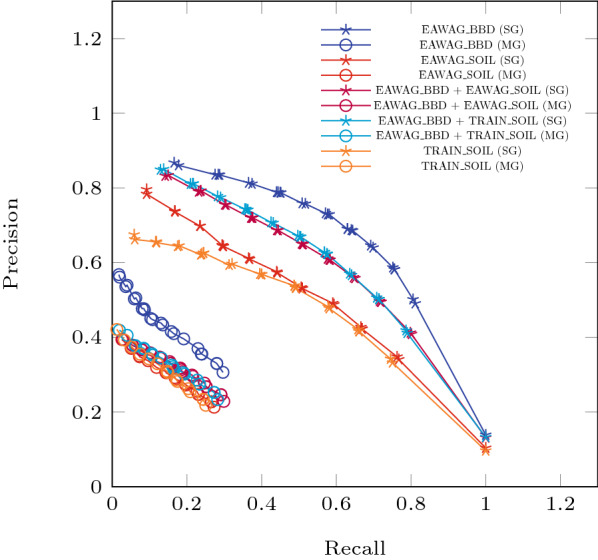
Precision-Recall curves for the *Evaluation with Holdout* experiments. The repeat-and-average component of this training approach quite effectively smooth out the kinks observed from the *Evaluation with Test Sets* experiments. The differences in the gap that indicates the compatibility between the transformation rules and the observed compound structures are more visible in the *Multi-Generation* results. While the lower numerical values simply reflect a different measuring standard in this new approach, the expected relationships between threshold, precision and recall are preserved

**Fig. 9:**
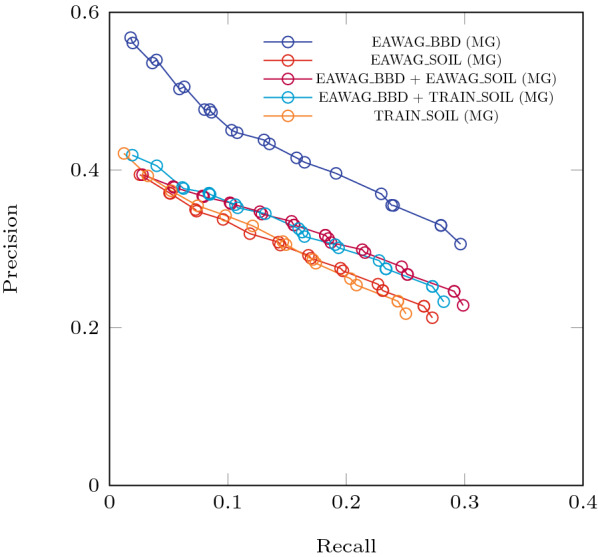
Precision-Recall curves from the *Multi-Generation* results for the *Evaluation with Holdout* experiments. The thresholds used for the curve are derived from the distribution of probability values from all reactions evaluated. Note that this plot is extracted from Fig. 8 to show the differences of the Multi-Generation performances

We trained Relative Reasoning models with the EAWAG-BBD package, EAWAG-SOIL package, TRAIN-SOIL package, EAWAG-BBD + EAWAG-SOIL, and EAWAG-BBD + TRAIN-SOIL packages. For all cases, we repeated a holdout evaluation 100 times. Tables 6 and 7 give the results, and Fig. 8 shows the associated Precision-Recall curves. A zoom-in to the *Multi-Generation* Precision-Recall curves from the *Evaluation with Holdout* experiments is presented in Fig. 9.

**Table 6:** Statistics of the *Evaluation with Holdout* experiments

Packages	Accuracy		Precision		Recall	
	SG	MG	SG	MG	SG	MG
BBD	0.65	0.17	0.58	0.31	0.76	0.3
SOIL	0.65	0.13	0.42	0.21	0.67	0.27
TRAIN$\_$SOIL	0.65	0.13	0.42	0.22	0.66	0.25
BBD+SOIL	0.62	0.15	0.49	0.22	0.72	0.3
BBD+TRAIN$\_$SOIL	0.63	0.15	0.5	0.233	0.71	0.28

**Table 7:** Statistics of the *Evaluation with Holdout* experiments

Packages	AUPRC		AUROC
	SG	MG	SG
BBD	0.64	0.12	0.87
SOIL	0.47	0.07	0.83
TRAIN$\_$SOIL	0.43	0.08	0.82
BBD+SOIL	0.56	0.09	0.85
BBD+TRAIN$\_$SOIL	0.57	0.09	0.85

Observations from the *Evaluation with Test Sets* results are also notably present in these results from a more repetitive averaging process. The Precision-Recall curves are also notably smoother from the averaging process. The curves from the *Multi-Generations* approach also distinguish more clearly between results from only using the EAWAG-BBD package and other configurations. This is partially due to the fact that transformation rules from the EAWAG-BBD package are used to train all of these relative reasoning models. These rules were optimized for EAWAG-BBD but not for the other packages for which they are less suitable. Also note that soil is a much more complex system compared to the typical in vitro culture studies, which EAWAG-BBD is mainly comprised of. The outcome of a degradation experiment in soil is more difficult to predict. [7]

Figure 7 and 8 indicate that the Precision-Recall curves from each of the evaluation approaches occupy a different region in this space. Numerical values of Precision and Recall from the *Multi-Generation* approach seem less ideal. However, it must be emphasized that this is not due to worse predictions (same prediction model), but from a more holistic evaluation, taking into account additional aspects on the pathway level.

In analogy to language processing, the *Single-Generation* approach is analogous to evaluating each predicted word written by a columnist individually. The *Multi-Generation approach* on the other hand, is analogous to extending this to sentences and paragraphs. That is, correct predictions in the former may be penalized in the latter for being in the wrong place. Such a relationship between the two approaches indicate that it is natural to expect this difference in resulting numerical values between the two approaches.

## Conclusion

In this paper, we present a new *Multi-Generation* approach for evaluating relative reasoning prediction models, that are used to predict biodegradation pathways. It includes methodology as well as performance in specifically designed experiments. The new approach evaluates predicted pathways with multiple generations of compounds holistically, in contrast to considering each reaction independently. Our approach additionally takes into consideration the increased uncertainty of observing compounds at higher depths in the pathways. We also propose an algorithm to account for intermediate metabolites, which would otherwise be incorrectly penalized during evaluation.

Our experiments show that the *Multi-Generation* evaluation metrics are much harder criteria. They provide a more realistic view on the prediction quality of whole pathways and reveal the incompleteness of the underlying transformation rules. With our new approach we can now start to compare the predictivity of different models on an objective basis in a model-agnostic way, i.e. independent from the model architecture and the set of transformation rules. Furthermore, we have demonstrated that our approach is more suitable to address two important characteristics of biotransformation pathway data: missing minor products in the reference data and intermediate metabolites. *Single-Generation* evaluation on the other hand might still be useful for determining the predictivity for individual (defined) transformation rules or for computationally demanding steps like hyper-parameter optimization.

Overall, our experiments demonstrate that it is still a long way until biotransformation prediction models can achieve top accuracy for complete pathways. However, with the *Multi-Generation* approach we improved our toolbox for the evaluation and comparison of pathway prediction models, which will facilitate the development of better models. In future work, we will use this approach to improve the compatibility of the biotransformation rules, for example by generating and testing new sets of rules. Additionally, we will integrate the new knowledge about likely intermediates into model training.

## Data Availability

The EAWAG-BBD [27] and EAWAG-SOIL [28] packages used in this study are publicly available on enviPath [29]. A python code that demonstrates the *Multi-Generation* evaluation process can be accessed at the enviPath Github repository [30], along with two files containing the names of pathways included in the *TRAIN-SOIL* and *TEST-SOIL* packages.

## Abbreviations

AUPRC: Area Under Precision-Recall Curve
AUROC: Area Under Receiver Operating Characteristic Curve
BBD: Biocatalysis/Biodegradation Database
BMPT: Biotransformer Metabolism Prediction Tool
BNICE: Biochemical network integrated computational explorer
CYP450: Cytochrome P450
EAWAG: Swiss Federal Institute of Aquatic Science and Technology (Eidgenössische Anstalt für Wasserversorgung, Abwasserreinigung und Gewässerschutz)
EC: Enzyme commission
FN: False negative
FP: False positive
KEGG: Kyoto Encyclopedia of Genes and Genomes
METEOR: Name of a knowledge-based expert system for toxicity and metabolism prediction
OASIS: Name of the company that manages the TIMES software
PPS: Pathway prediction system
RDM: Reaction center, the Difference region and the Matched region of the KEGG atom type changes
REACH: Registration, Evaluation, Authorization and Restriction of Chemicals (European Union Regulation)
TIMES: Name of the software from the Laboratory of Mathematical Chemistry Burgas for simulation of chemical metabolism
TN: True negative
TP: True positive
UM: University of Minnesota

## Footnotes

1 As the observed data is based on experiments where we can only be sure of observed compounds, we can never know for sure if a not observed compound is a truly negative or just not observed yet.
